# The effectiveness and safety of the rapid titration strategy of background controlled-release oxycodone hydrochloride for patients with moderate-to-severe cancer pain: A retrospective cohort study

**DOI:** 10.3389/fmed.2022.918468

**Published:** 2022-10-04

**Authors:** Weineng Feng, Yufeng Wang, Fengming Ran, Yong Mao, Helong Zhang, Qifeng Wang, Wen Lin, Zhidong Wang, Jianli Hu, Wangjun Liao, Tao Zhang, Qian Chu, Weijie Xiong, Tienan Yi, Jiqun Yi, Shoucheng Ma, Yi Sun, Lingzhan Meng, Chunling Liu, Silang Zhou, Dengyun Zheng, Shubin Wang, Haifeng Lin, Wenzheng Fang, Jun Li, Minhui Wu

**Affiliations:** ^1^Department of Head and Neck/Thoracic Medical Oncology, The First People's Hospital of Foshan, Foshan, China; ^2^Department of Cadre Medical Section, Yunnan Cancer Hospital & the Third Affiliated Hospital of Kunming Medical University & Yunnan Cancer Center, Kunming, China; ^3^Department of Oncology, Hubei Cancer Hospital, Wuhan, China; ^4^Department of Pain Management, Yunnan Cancer Hospital & the Third Affiliated Hospital of Kunming Medical University & Yunnan Cancer Center, Kunming, China; ^5^Department of Oncology, Tangdu Hospital of Air Force Medical University, Xi'an, China; ^6^Department of Thoracic Radiotherapy, Sichuan Cancer Hospital & Institute, Sichuan Cancer Center, Cancer Hospital Affiliate to School of Medicine, UESTC, Chengdu, China; ^7^Department of Oncology, Cancer Hospital of Shantou University Medical College, Shantou, China; ^8^Department of Oncology, Changsha Hospital of Traditional Chinese Medicine (Changsha Eighth Hospital), Changsha, China; ^9^Cancer Center, Xiehe Hosptial Tongji Medical College Huazhong University of Science and Technology, Wuhan, China; ^10^Department of Oncology, Nanfang Hospital, Southern Medical University, Guangzhou, China; ^11^Department of Oncology, The First Affiliated Hospital of Chongqing Medical University, Chongqing, China; ^12^Department of Oncology, Tongji Hospital, Tongji Medical College of Huazhong University of Science and Technology, Shanghai, China; ^13^Department of Oncology, Chengdu Fifth People's Hospital, Chengdu, China; ^14^Department of Oncology, The Central Hospital of Xiangyang, Chengdu, China; ^15^Department of Oncology, Guangzhou Red Cross Hospital, Guangzhou, China; ^16^Department of Oncology, The First Hospital of Lanzhou University, Lanzhou, China; ^17^Department of Hematology and Oncology, Hospital 452 of PLA, Chengdu, China; ^18^Department of Oncology, Chongqing Traditional Chinese Medicine Hospitai & No. 4 Clinical Medicine School of Chengdu University of TCM, Chongqing, China; ^19^Respiratory Department, Xinjiang Cancer Hospital, Urumqi, China; ^20^Department of Oncology, PLA Army 74 Group Army Hospital, Guangzhou, China; ^21^Department of Oncology, Jinshazhou Hospital of Guangzhou University of Chinese Medicine, Guangzhou, China; ^22^Department of Oncology, Peking University Shenzhen Hospital, Shenzhen, China; ^23^Department of Oncology, The Second Affiliated Hospital of Hainan Medical University, Haikou, China; ^24^Department of Oncology, Fuzhou PLA General Hospital, Fuzhou, China; ^25^Department of Oncology, The Central Hospital of Wuhan, Wuhan, China; ^26^Department of Integrated Chinese and Western Medicine, Shaanxi Cancer Hospital Affiliated to Medical College of Xi'an Jiaotong University, Xi'an, China

**Keywords:** immediate-release (IR) morphine, controlled-release (CR) oxycodone, cancer pain, dose titration, oxycodone hydrochloride, pain remission

## Abstract

**Background:**

Oxycodone hydrochloride is a semisynthetic narcotic analgesic agent. This study aimed to explore optimal titration strategy of controlled-release (CR) oxycodone hydrochloride in patients with cancer pain.

**Methods:**

258 patients, who used regular strong opioids (morphine and CR oxycodone hydrochloride) for cancer pain across 25 three grade class hospitals in China during January 15th 2017 to April 30th 2017, were retrospectively studied. The patients were divided into 4 groups according to treatment regimens titrated. The pain remission rate and numeric rating scale (NRS) of cancer pain was recorded at 0, 12, 24, 36, 48, 60, 72 h after opioid titration. The incidence of adverse events (AEs) with therapy were also observed.

**Results:**

12 h after treatment, pain remission rate of Group B, C and D was significantly higher (*P* < 0.001) than Group A. For the complete remission rate, there were also significant differences among the four groups (*P* < 0.001). No significant difference was found among four groups for pain remission rate at 24, 72 h after treatment. Multiple comparison of NRS scores showed that the both Group B and C varied significantly with Group D (*P* = 0.028, *P* = 0.05, respectively), showing superior analgesic effect over Group D. AEs were significantly different among groups (*P* < 0.01), with the most frequent AEs in Group A, lowest in Group B.

**Conclusion:**

The rapid titration strategy of background CR oxycodone hydrochloride was effectiveness and safety in patients with moderate-to-severe cancer pain.

## Introduction

According to World Health Organization (WHO) guidelines, the dose of opioid drugs can be titrated upward in management of chronic, moderate to severe cancer pain, until an acceptable balance between analgesia and side effects. Hence, the initiation and titration of an opioid for moderate to severe pain is a critical point during individual patient treatment. According to the guidelines of the European Society for Medical Oncology (EMSO), opioid should be titrated as rapidly as possible to take effect. Morphine remains the traditional choice for opioid agonists. However, it may be difficult to find an optimal initial dose, due to the great variation of oral bio-availability of morphine (15–64%) in individual patient, and relatively high incidence of side effects, especially in treatment beginning (1). Controlled release (CR) oxycodone is an easily administered opioid with analgesia effects lasting up to 12 h (2).

Oxycodone hydrochloride is a semisynthetic narcotic analgesic agent. It has been used since 1917 with a clinical impression, due to it is as effective as morphine, with fewer side effects. Oxycodone is also available in a single-entity CR formulation for treatment of moderate to severe pain. CR opioid formulation offers advantage of convenient dosing schedule for around-the clock analgesia. However, it has significantly longer duration of pain remission than that provided by the immediate-release (IR) formulation (3). In addition, CR oxycodone has been shown to be as effective as CR morphine in treatment of chronic cancer pain (4–6). Hence, CR oral oxycodone may likewise be used for titration to stable pain control as readily as morphine.

IR morphine has been recommended in a dose titration procedure to achieve rapid onset during pain management (7). Then, it is switched to CR formulation for maintenance therapy when titrated up to stable pain control (8). While this 4-hourly scheduled procedure is cumbersome and inevitably, causing problems of compliance of patients and physicians (9, 10). Meanwhile, the increased dose of morphine may expose patients to high incidence of adverse effects (7). Oxycodone may omit the need for opioid switching from IR morphine to a controlled-release preparation. Dose titration of oral CR and IR oxycodone has been suggested as one of the titration schedules for moderate and severe cancer pain (11). However, few studies have focused on the effect of background treatment with CR oxycodone on dose titration during pain management.

There was no consensus regarding the dose titration of oxycodone. When titrating with background CR oxycodone, many clinicians adjusted the subsequent dose, according to the frequency of pain episodes, rescue medication, or the intensity of pain in previous 12 h after treatment. In order to quickly adjust the opioid to appropriate dose and quickly relieve the patient's pain, this study adjusted the amount of CR oxycodone to 12 h after the first dose. Therefore, this retrospective study aimed to identify the optimal titration strategy of CR oxycodone as background dose to achieve adequate pain remission in patients with moderate to severe cancer pain.

## Methods and materials

### Study population

Two hundred and fifty-eight patients, who used regular strong opioids (morphine and CR oxycodone hydrochloride) for cancer pain across 25 three grade class hospitals in China during January 15th 2017 to April 30th 2017, were retrospectively study. The patients were eligible for enrolment if they (1) aged 18–80 years; (2) histologically or cytologically confirmed diagnosis of cancer; (3) moderate to severe pain with Numeric Pain Scale Ratings (NRS) ≥4; (4) had ability to understand and communicate with the doctor regarding the treatment regimen. Patients were excluded if they (1) had neuropathic pain; (2) was pregnant or breastfed in women with childbearing potential; (3) had an oncologic emergency; (4) showed some contraindication to the use of strong opioid; (5) major liver and kidney function impairment [aminotransferase (AST), alanine aminotransferase (ALT) Creatinine or urea nitrogen ≥ thrice of upper limit of normal]; (6) had a history of alcohol or drug abuse.

### Study design and treatment regimen

The administration of baseline opioids for pain management was checked in each case, and patients were divided into 4 groups according to treatment regimens.

#### Group A

Patients were initially titrated with IR morphine 4 hourly, and were reassessed according to response of the patients and performed dose titration every 60 min. The starting dose was determined by the treating physician according to international guidelines, and titrated until adequate pain control was achieved. At this stage, patients were switched to equianalgesic oxycodone.

#### Group B

For opioid-naïve patients, whose pain intensity is moderate or severe, the doses of oxycodone hydrochloride CR tablets (OxyContin^®^ Tablets, Bard Pharmaceuticals Limited, UK) were 10 or 20 mg, respectively, every 12 h. For opioid-tolerant patients, the initial dose of oxycodone was equivalent to 1/2 of total opioid taken in the previous 24 h. When the frequency of rescue medication ≥2 in the previous 12 h, 10 mg of oxycodone was added to the current regular dose at 12, 24, 36, 48, 60, and 72 h after treatment. When the frequency of breakthrough pain episodes < 2 in the previous 12 h, the current dose was repeated.

#### Group C

The subsequent dose at 12, 24, 36, 48, 60, and 72 h after treatment was adjusted as the total amount of rescue doses (converted to oxycodone equivalent dose) taken in the previous 12 h plus initially regularly scheduled oxycodone dose.

#### Group D

When the intensity of breakthrough pain was ever higher than 7 (NRS) in the previous 12 h, the subsequent dose was increased by 50–100% at 12, 24, 36, 48, 60, and 72 h after treatment. If higher than 4 (NRS), the subsequent dose was increased by 25–50%.

### Data collection

Information was gathered from patients' electronic medical record, including general demographic data (age and gender), cancer status (site and metastasis), analgesic therapy, NRS score at 0, 12, 24, 36, 48, 60, and 72 h after opioid titration.

The pain intensity of each patient was evaluated by a numeric rating scale from 0 to 10, in which 0 indicates no pain at all, and 10 indicates the worst imaginable pain that cannot be tolerated. Pain remission rate was defined as the rate of patients with NRS score ≤ 3 [CR (Complete remission)] or achieving a reduction of 25% when NRS score >3 [PR (Partial remission)] at 24 h.

The pain remission rate at 12, 24, and 72 h was evaluated. In addition, the NRS score during 72 h after opioid titration, the frequency of rescue medication during opioid titration period, and the incidence of clinical side effects associated with therapy, including constipation, nausea, and dizziness, were also observed.

### Statistical analysis

Statistical analyses were performed using SPSS, version 17.0 (SPSS, Inc., Chicago, IL). Baseline comparisons were performed using chi-square test or Fisher's exact test for categorical variables and *t*-test or non-parametric Mann–Whitney *U* test for continuous variables. We examined the primary aim, using a linear mixed model for repeated measures. For outcomes with significant difference at baseline, it was adjusted for confounders to obtain the estimated marginal means (EMM) with standard error (SE). A *P-*value of < 0.05 was considered as statistically significant.

## Results

### Clinical characteristics

A total of 258 cancer patients were included in this retrospective study. Demographics of the patient population are shown in Table 1. There were 18 patients in Group A, 109 in Group B, 43 in Group C and 88 in Group D. No statistically significant differences between four groups with respect to age, gender, pre-treatment analgesics, tumor type and the use of adjuvant analgesic (*P* > 0.05). However, it showed significant differences with respect to smoking, bone metastasis, pain pathology and baseline NRS score among group (*P* ≤ 0.001). Correlation analysis showed that smoking, bone metastasis and pain pathology were associated with NRS score.

**Table 1:** Patient demographics and baseline clinical characteristics.

**Characteristic**	**Group A (*n* = 18)**	**Group B (*n* = 109)**	**Group C (*n* = 43)**	**Group D (*n* = 88)**	***P***
Age (years)	60.5 ± 11.8	58.2 ± 9.9	57.1 ± 9.1	57.4 ± 12.1	0.669
Male/female (cases)	12/6	78/31	25/18	49/39	0.107
Smoker (*n*, %)	12 (66.7)	43 (39.8)	21 (48.8)	14 (16.7)	< 0.001
Drinker (*n*, %)	2 (11.1)	24 (22.2)	14 (32.6)	11 (13.1)	0.056
Pre-treatment analgesics	11 (61.1)	70 (64.8)	25 (58.1)	58 (69.0)	0.83
Tumor type (*n*, %)					0.145
Breast	1	4	2	6	
Lung	13	56	25	23	
Colon/rectum	1	7	4	11	
Head/neck	1	13	2	9	
Pancreas/stomach	1	7	2	5	
Others	1	22	8	34	
Bone metastasis (*n*, %)	1 (5.6%)	29 (26.6%)	9 (20.9%)	16 (18.2%)	0.001
Adjuvant analgesic (*n*, %)	0	0	0	3 (3.4)	0.241
Pain pathological (*n*, %)					0.001
Nociceptive	15 (83.3%)	75 (68.8%)	29 (67.4%)	39 (44.3%)	
Mixed	3 (16.7%)	34 (31.2%)	14 (32.6%)	49 (55.7%)	
Baseline NRS score	4.33 ± 0.97	5.32 ± 1.19	5.56 ± 1.69	6.09 ± 1.26	< 0.001

### Pain remission rate during titration period

After treatment for 12 h, pain remission rate of Group B (77.06 %), Group C (65.12%), and Group D (59.77%) was significantly higher (*P* < 0.001) than Group A (22.22 %, Table 2). For the complete remission rate, there were also significant differences among the four groups (*P* < 0.001), and complete remission rate of Group B (69.72%), Group C (58.14%) was significantly higher than Group A (22.22 %) (*P* = 0.0022), and complete remission rate of Group B was also significantly higher than Group D (43.18%, *P* < 0.001). After treatment for 24 h, 85.0% (204/240) patients titrated with background CR oxycodone had pain remission, and 71.4% (13/18) patients titrated with IR morphine had pain remission. Among the four groups, no significant difference was found in term of pain remission rate at 24 h (71.4 % Group A, 87.5% for Group B, 86.8% for Group C, and 82.1% for Group D, *P* = 0.373). However, there were significant differences in complete remission rate among the four groups (*P* = 0.049). Patients who titrated with CR oxycodone as baseline opioid in group B had a higher rate of complete remission than patients in group D (*P* = 0.007) (Table 2). After treatment for 72 h, no significant difference was found among the four groups in terms of pain remission rate or complete remission rate (94.44%, 83.33% for Group A, 94.44%, 92.66% for Group B, 95.35%, 93.02% for Group C, and 96.51%, 90.91% for Group D, all *P* > 0.05).

**Table 2:** Pain remission rates at 12 h after treatment, and complete pain remission rates at 24 h after treatment.

**12 h after treatment**	**Pain remission**	**No remission**	**χ^2^**	***P***
Group	*n* (%)	*n* (%)	22.59	< 0.001
A	4 (22.22)	14 (77.78)	–	–
B	84 (77.06)	25 (22.94)	21.836	< 0.001*
C	28 (65.12)	15 (34.88)	9.361	0.0022*
D	52 (59.77)	35 (40.23)	8.448	0.0037*
**24 h after treatment**	**Complete remission**	**No complete remission**	**χ^2^**	***P***
Group	*n* (%)	*n* (%)	7.87	0.049^#^
A	12 (66.67)	6 (33.33)		
B	91 (83.49)	18 (16.51)		
C	32 (74.42)	11 (25.58)		
D	59 (67.05)	29 (32.95)	7.245	0.007^Δ^

^*^Compared with group A.

^Δ^Comparison between group B and group D.

^#^Comparison among four groups.

### NRS score during titration period

**Figure 1:**
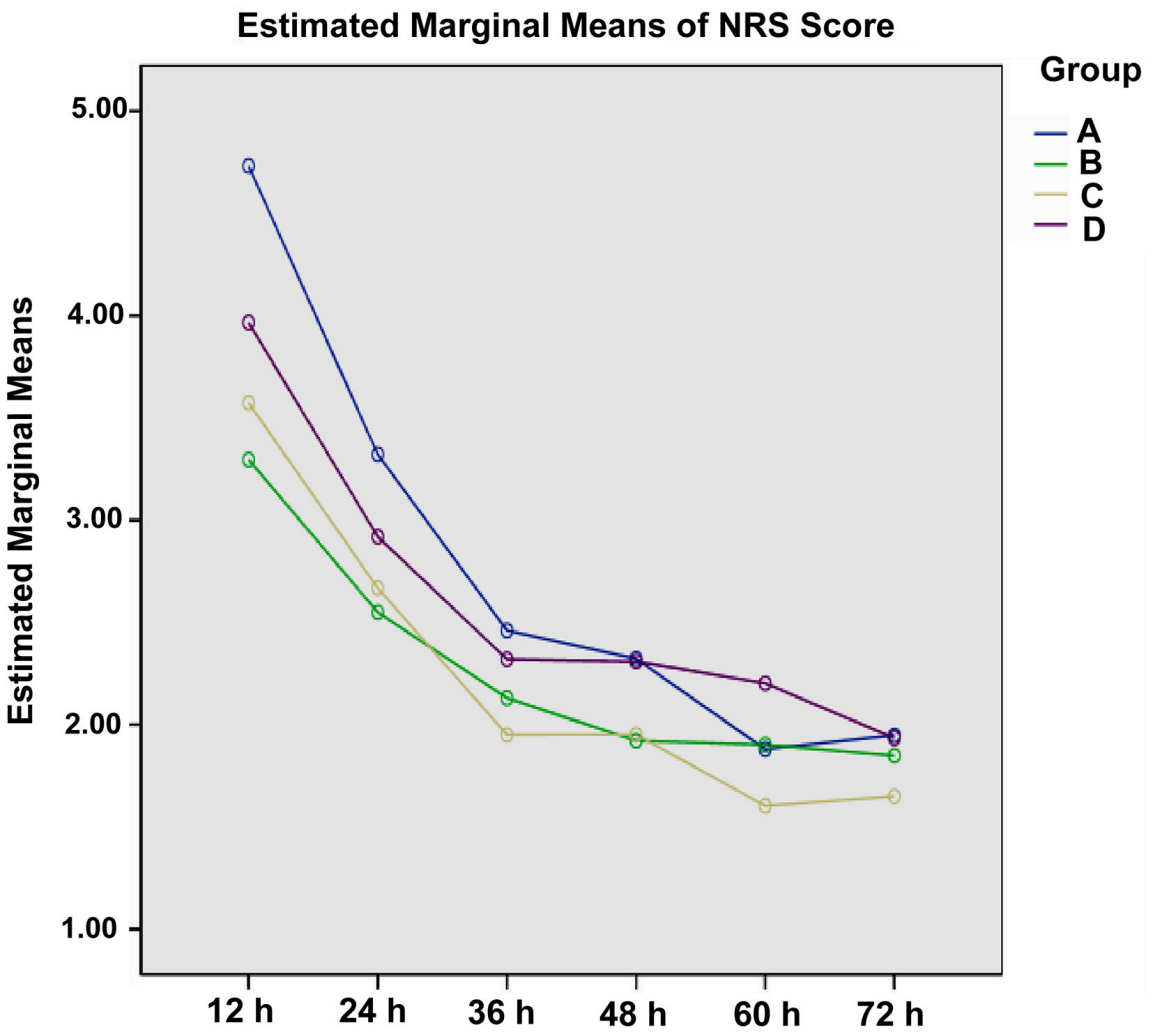
Estimated marginal means for the NRS score of four titration strategies at each data collection period.

Considering the significant difference of NRS score at baseline (*P* < 0.05), covariance analysis was performed. The NRS score in each group showed a decline trend over time (Table 3). For each group, the opioid titration had a beneficial effect on pain outcomes. After administrating opioid analgesics 24 h, estimated marginal means for NRS scores were < 3 for treatment regimens titrated with background CR oxycodone [2.549 ± 0.148; 95% CI (2.257, 2.841)] for Group B, 2.668 ± 0.234 [95% CI (2.208, 3.128)] for Group C, and 2.919 ± 0.172 [95% CI (2.580, 3.258)] for Group D. While, for the treatment regimen titrated with IR morphine, estimated marginal means for the NRS score was 3.322 ± 0.372 [95% CI (2.589, 4.054)] for Group A. Estimated marginal means of NRS score in four groups at each time period are shown in Figure 1. During the titrating period, the downward trend was more stable in Group B, with a little fluctuation in Group A, Group C, and Group D. Result of multiple comparison of NRS scores after opioid titration at different data collection period showed that both Group B and Group C varied significantly with Group D (Group B vs. Group D, *P* = 0.028; Group C vs. Group D, *P* = 0.05), and had a superior analgesic effect over Group D. The superiority of Group B and Group C also could be observed from estimated marginal means for the NRS score when compared Group A (2.777 ± 0.245 for Group A, 2.274 ± 0.098 for Group B, 2.232 ± 0.154 for Group C, and 2.608 ± 0.113 for Group D), and the difference did not attain statistical significance (*P* = 0.055, 0.061).

**Table 3:** Estimated marginal means for NRS score at 12, 24, 36, 48, 60, and 72 h after treatment.

**Covariates** ^**a**^		**Group A**	**Group B**	**Group C**	**Group D**
12 h	Mean ± S.E.	4.733 ± 0.429 (3.889, 5.557)	3.296 ± 0.171 (2.959, 3.633)	3.574 ± 0.269 (3.043, 4.104)	3.967 ± 0.198 (3.576, 4.357)
	95% CI				
24 h	Mean ± S.E.	3.322 ± 0.372 (2.589, 4.054)	2.549 ± 0.148 (2.257, 2.841)	2.668 ± 0.234 (2.208, 3.128)	2.919 ± 0.172 (2.580, 3.258)
	95% CI				
36 h	Mean ± S.E.	2.460 ± 0.306 (1.858, 3.063)	2.13 ± 0.122 (1.889, 2.370)	1.950 ± 0.192 (1.572, 2.329)	2.320 ± 0.142 (2.041, 2.598)
	95% CI				
48 h	Mean ± S.E.	2.322 ± 0.304 (1.723, 2.921)	1.921 ± 0.121 (1.682, 2.160)	1.951 ± 0.191 (1.575, 2.326)	2.308 ± 0.141 (2.031, 2.585)
	95% CI				
60 h	Mean ± S.E.	1.879 ± 0.292 (1.303, 2.455)	1.901 ± 0.117 (1.671, 2.131)	1.602 ± 0.184 (1.241, 1.964)	2.202 ± 0.135 (1.935, 2.468)
	95% CI				
72 h	Mean ± S.E.	1.948 ± 0.269 (1.418, 2.477)	1.848 ± 0.107 (1.637, 2.059)	1.649 ± 0.169 (1.316, 1.981)	1.934 ± 0.124 (1.689, 2.179)
	95% CI				

^a^Covariates appearing in the model are evaluated at the following values: The NRS score = 5.5415 before treatment.

In order to explore the correlation between pain pathology and the effectiveness of opioid analgesia, we analyzed the relationship between pain pathology and NRS score. It found that compared to the nociceptive pain group, the mixed pain group was associated with higher NRS score (*P* < 0.001) (Supplementary Figure 1). Therefore, the significantly higher proportion of patients with mixed pain in Group D (55.7%) may partially explain the higher NRS score compared to Group B and Group C (31.2%, 32.6%) (*P* < 0.001).

### The frequency of rescue medication

Among the four groups, there was no significant difference in terms of the frequency of rescue medication during 0–12, 12–24, 24–36, 36–48, 48–60, and 60–72 h after treatment, respectively. While, except for Group A, there were significant difference in term of the frequency of rescue medication among different time periods after treatment (0–12, 12–24, 24–36, 36–48, 48–60, and 60–72 h) in group B, C and D (P < 0.05), and showed an overall declining tendency as administration time extended (Table 4). At 24 h after administrating opioid analgesics, the frequency of rescue medication was 44.4% for Group A, 29.4% for Group B, 20.9% for Group C, and 25.0% for Group D. The proportion of patient experiencing rescue medication at 72 h after treatment was 22.2, 8.3, 14.0, 11.4%, respectively.

**Table 4:** The frequency of rescue medication at 12, 24, 36, 48, 60, and 72 h after treatment.

**Groups**	**12 h**	**24 h**	**36 h**	**48 h**	**60 h**	**72 h**	***P* ^α^**
A	33.3% (6/12)	44.4% (8/10)	22.2% (4/14)	27.8% (5/13)	16.7% (3/15)	22.2% (4/14)	0.54
B	48.6% (53/56)	29.4% (32/77)	18.3% (20/89)	15.6% (17/92)	10.1% (11/98)	8.3% (9/10)	< 0.000
C	44.2% (19/24)	20.9% (9/34)	16.3% (7/36)	14.0% (6/37)	2.3% (1/42)	14.0% (6/37)	0.011
D	55.7% (49/39)	25.0% (22/66)	15.9% (14/74)	17.0% (15/73)	12.8% (10/78)	11.4% (10/78)	< 0.000
P^β^	0.295	0.274	0.885	0.581	0.192	0.279	

^α^Intra group comparison.

^β^Comparison among four groups.

### Adverse events during opioid titration

Of the 390 total AEs that occurred during open-label titration, most (96.2%) were mild or moderate in intensity. Severe adverse events occurred in 16 (4.1%) patients. A summary of AEs according to successful titration is presented in Table 5. Constipation, dizziness, and nausea were the most frequent treatment-related AEs. The occurrence of AEs significantly differed among groups (*P* < 0.0001), and Group A has the most frequent AEs.

**Table 5:** Adverse events associated with the four treatment regimens.

**Adverse event,** ***n* (%)**	**Group A (*n* = 18)**	**Group B** **(*n* = 109)**	**Group C (*n* = 43)**	**Group D (*n* = 88)**	***P***
Constipation					< 0.0001
Mild	6 (33.3%)	37 (33.9%)	23 (53.5%)	35 (39.8%)	
Moderate	10 (55.6%)	38 (34.9%)	11 (25.6%)	19 (21.6%)	
Severe	1 (5.6%)	3 (2.8%)	4 (9.3%)	5 (5.7%)	
Nausea					< 0.0001
Mild	11 (61.1%)	29 (26.6%)	25 (58.1%)	24 (27.3%)	
Moderate	5 (27.8%)	11 (10.1%)	0 (0%)	4 (4.5%)	
Severe	0 (0%)	1 (0.9%)	0 (0%)	1 (1.1%)	
Dizziness					< 0.0001
Mild	15 (83.3%)	39 (35.8%)	30 (69.8%)	27 (30.7%)	
Moderate	1 (5.6%)	8 (7.3%)	2 (4.7%)	5 (5.7%)	
Severe	0 (0%)	0 (0%)	0 (0%)	1 (1.1%)	
Other adverse event	0 (0%)	2 (1.8%)	0 (0%)	6 (6.8%)	0.15

## Discussion

The European Association for Palliative Care (EAPC) guidelines recommended that IR and slow-release oral formulations of morphine, oxycodone, and hydromorphone can be used for dose titration. The titration schedules for both types of formulation should be supplemented with oral IR opioids given as needed (11). In term of scheduling and titration, EMSO Clinical Practice guidelines recommended that opioid doses should be titrated to take effect as rapidly as possible. It's worth noting that, in our study, the dose of oxycodone hydrochloride was adjusted every 12 h, while for the traditional titration strategies, the dose was adjusted at 24 h. The previous studies have shown that the 12 h titration scheme of oxycodone hydrochloride could achieve better analgesic effect compared to 24 h titration scheme (12). The potential reasons are as follows, on one hand, the adjustment was consistent with the sustained action of hydroxycodone, which continuous analgesia for 12 h (13); on the other hand, patients with moderate and severe cancer pain do not need endure the pain of adjusting the dose for 24 h, so that the patients could get pain remission more quickly. In addition, the 12 h titration scheme may alleviate the shortage of medical resources in developing countries to a certain extent.

In this study, with the 12 h titration scheme, we compared four titration strategies of CR oxycodone or IR morphine as background dose to achieve adequate pain remission in patients with moderate to severe cancer pain. For each group, the opioid titration had a beneficial effect on pain outcomes. The NRS score in each group showed a decline trend over time. After treatment for 12 h, pain remission rate of patients who titrated with CR oxycodone as baseline opioid (77.06% for Group B, 65.12% for Group C, and 59.77% for Group D) was significantly higher (*P* < 0.001) than that titrated with IR morphine (22.22 % for Group A). This means that CR oxycodone could achieve rapid analgesic effect (2).

Clinical outcomes of opioids titration for cancer pain are particularly critical on the first day, and the rapid and stable analgesia can not only reduce the pain symptoms of patients, but also increase the patient confidence in the subsequent treatment. In this study, at 24 h after treatment, 84.8% (219/258) of the patients had pain remission, and 75.2% (194/258) of the patients had complete remission. Wasaburo Koizumi et al. have reported that the pain remission rate at 24 h after the initial CR oxycodone intake (starting with a 5 mg CR oxycodone every 12 h and the dose could be titrated against the intensity of pain) was 70% (95% CI 45.7–88.1) (14). The overall pain remission rate at 24 h after treatment appears higher than the previous results which adjusted the CR oxycodone hydrochloride dose every 24 h. In each group, most of the patients obtained pain remission during the study, but there were significant differences in complete remission rate among the four groups at 24 h after treatment (*P* = 0.049). Patients who titrated with CR oxycodone as baseline opioid in group B had a higher rate of complete remission than patients in group D (*P* = 0.007). Opioids are the current standard treatment of moderate or severe nociceptive pain, while neuropathic pain or mixed in origin is generally considered to be a little resistant to opioids (15). Result of multiple comparison of NRS scores after opioid titration at different data collection period showed that both Group B and Group C varied significantly with Group D (Group B vs. Group D, *P* = 0.028; Group C vs. Group D, *P* = 0.05), and had a superior analgesic effect over Group D, which may be attributed to the significantly higher proportion of patients with mixed pain in Group D (55.7%) than that of Group B and Group C (31.2%, 32.6%) (*P* < 0.001). The superiority of Group B and Group C can also be observed from estimated marginal means for the NRS score when compared with Group A, and the difference did not attain statistical significance (*P* = 0.055, *P* = 0.061, respectively).

Nevertheless, side effects such as emesis, sedation, hallucinations constipation, nausea and dizziness have been described following morphine administration, while being less frequent at equianalgesic doses after oxycodone (4, 5). In this study, Chi-square analyses indicated constipation, nausea and dizziness differed significantly among groups (*P* < 0.01), with the most frequent AEs in Group A. There are two major advantages in adjusting the CR oxycodone hydrochloride dose every 12 h. On one hand, titration with IR morphine for breakthrough cancer pain exist major limitations. This method are the intricate multiple dose schedule, including the nighttime or double bedtime doses, the appropriateness of IR morphine for breakthrough pain and the need to modify drug formulation as well as administration timing once titration to pain remission is reached (16–18). These complex processes may reduce patient compliance and poor adherence to the therapy regimen is a major barrier to effective cancer pain management (19). The use of CR oxycodone can not only overcome the disadvantage of poor compliance, but also alleviate the shortage of medical resources in developing countries to a certain extent. On the other hand, adjusting the amount of CR oxycodone to 12 h could quickly adjust the opioid to appropriate dose and quickly relieve the patient's pain.

The main limitations of this report are its retrospective and observational nature, relatively short duration and small sample size of group A. Because of the retrospective design, randomization was not possible. Thus, unlike randomized and controlled studies, this observational study was intended to convey only exploratory analysis and not to confirm or reject a hypothesis. Based on this study, a new randomized and large-scale clinical studies to confirm the treatment efficacy can now be more carefully planned.

## Conclusion

For patients with moderate-to-severe cancer pain, the rapid titration strategy of background CR oxycodone hydrochloride was more effective and tolerant than IR morphine. The dosage adjustment protocol according to the frequency of breakthrough pain episodes or rescue medication (Group B), or the total amount of rescue doses (Group C) may provide better analgesia than that according to the intensity of breakthrough pain (Group D). It appears that adjusting the CR oxycodone hydrochloride dose every 12 h may achieve rapid and stable analgesic effects. A large-scale clinical trial on rapid pain control is need in the future to find a better CR background titration strategy that provide more clinical benefit for cancer patients.

## Data availability statement

The original contributions presented in the study are included in the article/Supplementary material, further inquiries can be directed to the corresponding author/s.

## Ethics statement

Ethical review and approval was not required for the study on human participants in accordance with the local legislation and institutional requirements. The patients/participants provided their written informed consent to participate in this study. Written informed consent was obtained from the individual(s) for the publication of any potentially identifiable images or data included in this article.

## Author contributions

WeiF and YW conceived the study. FR, YM, HZ, QW, WLin, ZW, JH, WLia, TZ, QC, and WX performed the literature search and writing of the manuscript. TY, JY, SM, YS, LM, CL, and SZ analyzed and interpreted the data. DZ, SW, HL, WenF, and JL collected and assembled the data. MW submitted the manuscript and is the corresponding author. All authors read and approved the final manuscript.

## Conflict of interest

The authors declare that the research was conducted in the absence of any commercial or financial relationships that could be construed as a potential conflict of interest.

## Publisher's note

All claims expressed in this article are solely those of the authors and do not necessarily represent those of their affiliated organizations, or those of the publisher, the editors and the reviewers. Any product that may be evaluated in this article, or claim that may be made by its manufacturer, is not guaranteed or endorsed by the publisher.

## Supplementary material

Supplementary Figure 1 Estimated marginal means for the NRS Score between nociceptive and mixed pain group at each data collection period.
