# Supplementary figures and images for: The effectiveness and safety of the rapid titration strategy of background controlled-release oxycodone hydrochloride for patients with moderate-to-severe cancer pain: A retrospective cohort study
Source: Front Med (Lausanne). 2022 Oct 4;9:918468. doi: 10.3389/fmed.2022.918468 (PMC9576945; doi:10.3389/fmed.2022.918468)

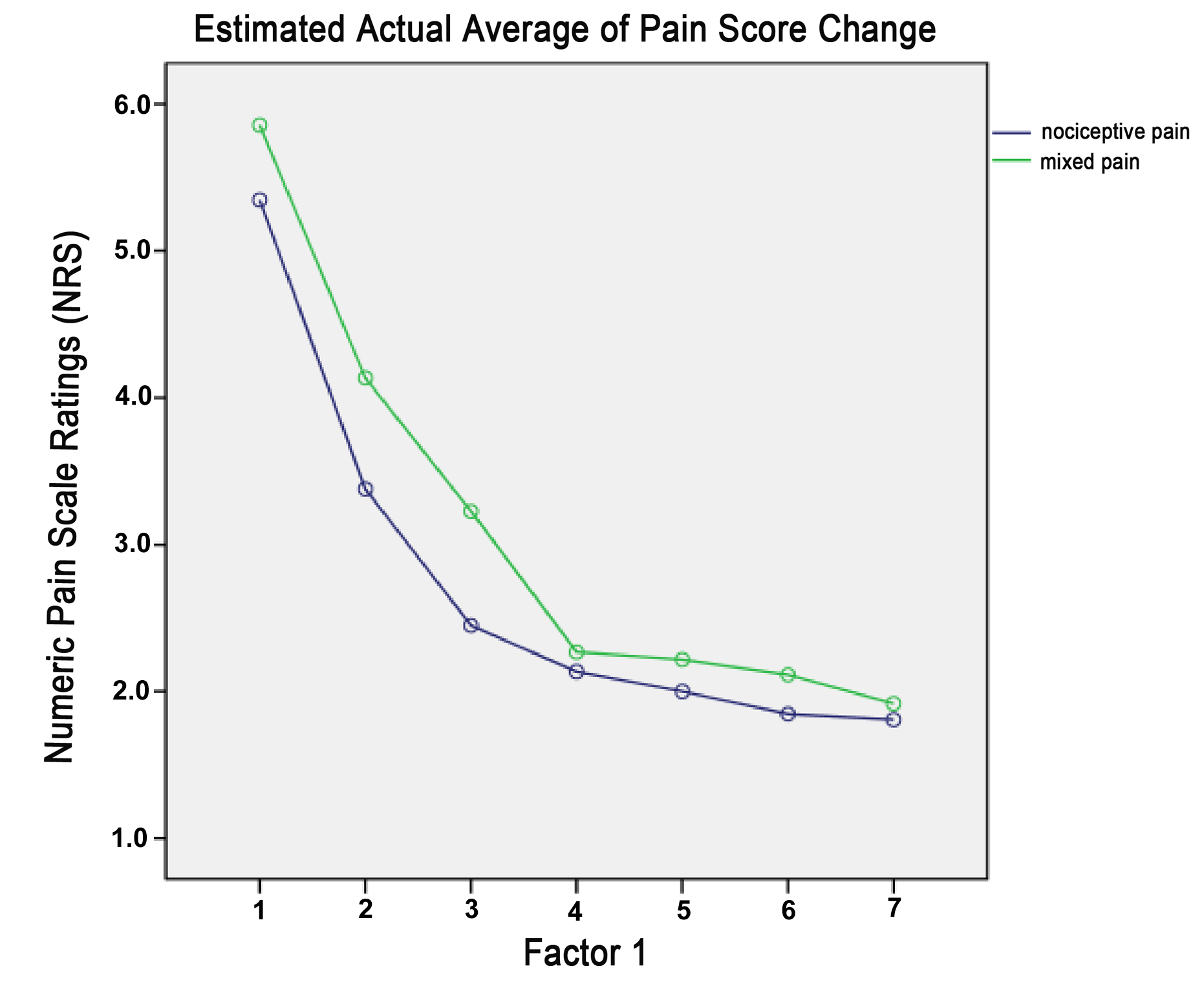

Supplement: Supplementary Figure 1 — Estimated marginal means for the NRS Score between nociceptive and mixed pain group at each data collection period. [file Image_1.TIF]
